# Freshwater exchanges and surface salinity in the Colombian basin, Caribbean Sea

**DOI:** 10.1371/journal.pone.0182116

**Published:** 2017-08-04

**Authors:** Emilio Beier, Gladys Bernal, Mauricio Ruiz-Ochoa, Eric Desmond Barton

**Affiliations:** 1 Centro de Investigación Científica y de Educación Superior de Ensenada–Unidad La Paz, La Paz, Baja California Sur, México; 2 Departamento de Geociencias y Medio Ambiente, Universidad Nacional de Colombia, Medellín, Colombia; 3 Programa de Ingeniería Ambiental, Facultad de Ingeniería, Universidad Manuela Beltrán, Bucaramanga, Colombia; 4 Departamento de Oceanografía Instituto Investigaciones Marinas (CSIC), Vigo, España; University of California San Diego, UNITED STATES

## Abstract

Despite the heavy regional rainfall and considerable discharge of many rivers into the Colombian Basin, there have been few detailed studies about the dilution of Caribbean Surface Water and the variability of salinity in the southwestern Caribbean. An analysis of the precipitation, evaporation and runoff in relation to the climate variability demonstrates that although the salt balance in the Colombian Basin overall is in equilibrium, the area south of 12°N is an important dilution sub-basin. In the southwest of the basin, in the region of the Panama-Colombia Gyre, Caribbean Sea Water is diluted by precipitation and runoff year round, while in the northeast, off La Guajira, its salinity increases from December to May by upwelling. At the interannual scale, continental runoff is related to El Niño Southern Oscillation, and precipitation and evaporation south of 12°N are related to the Caribbean Low Level Jet. During El Niño years the maximum salinification occurs in the dry season (December-February) while in La Niña years the maximum dilution (or freshening), reaching La Guajira Coastal Zone, occurs in the wet season (September-November).

## Introduction

The Caribbean Sea is considered a concentration basin (i.e. evaporation exceeds precipitation) even though the increase of salinity is not enough to cause deep vertical convection [1–3]. Schmidt et al. [4] concluded that Caribbean salinity is not significantly affected by freshwater runoff and primarily reflects the evaporation/precipitation ratio. On the other hand, Yoo and Carton [2] noted that the river discharge and the annual average rate of advection of freshwater into the Caribbean Sea from the Atlantic differ by just over 10%.

The transport of water through the succession of Grenada, Venezuelan, Colombian, Cayman and Yucatan basins that constitute the Caribbean forms part of the western boundary current system of the North Atlantic Ocean and therefore is associated with the global meridional overturning circulation. Surface currents in the upper 50-to-100 m layer transport Caribbean Surface Water (CSW), which is formed by dilution of Atlantic Water by freshwater discharge mainly from the Amazon and Orinoco Rivers as it is transported into the Caribbean Sea by the Guiana Current [5–7]. Recently, Grodsky et al. [8] investigated the propagation of interannual changes in the Amazon plume as salinity anomalies through the central Caribbean on into the Gulf Stream system. CSW is transported by the Caribbean Current, which accelerates toward the Yucatan Channel, and has an average speed greater than 25 cm s^-1^ [9–11]. The Caribbean Current is made up of two cores that flow westward through the Venezuelan Basin to join in the Colombian Basin. As in all Caribbean sub-basins, the flow is strongly influenced by coherent eddy motions with horizontal scales of 100 km [7, 10, 12–14]. Salinity anomalies propagate westward with these eddies, at an average speed of 11 cm s^-1^ [8]. In the southwest Caribbean, roughly south of 12°N, a recirculation takes place in the cyclonic Panama-Colombia Gyre as described by Mooers and Maul [15] and Richardson [10], among others. The southern limb of the Panama-Colombia Gyre constitutes the nearshore Panama-Colombia Countercurrent, which near 75°W mainly turns back with the general westward flow, though a proportion continues eastward along the continental slope as an undercurrent [12, 16].

**Fig 1:**
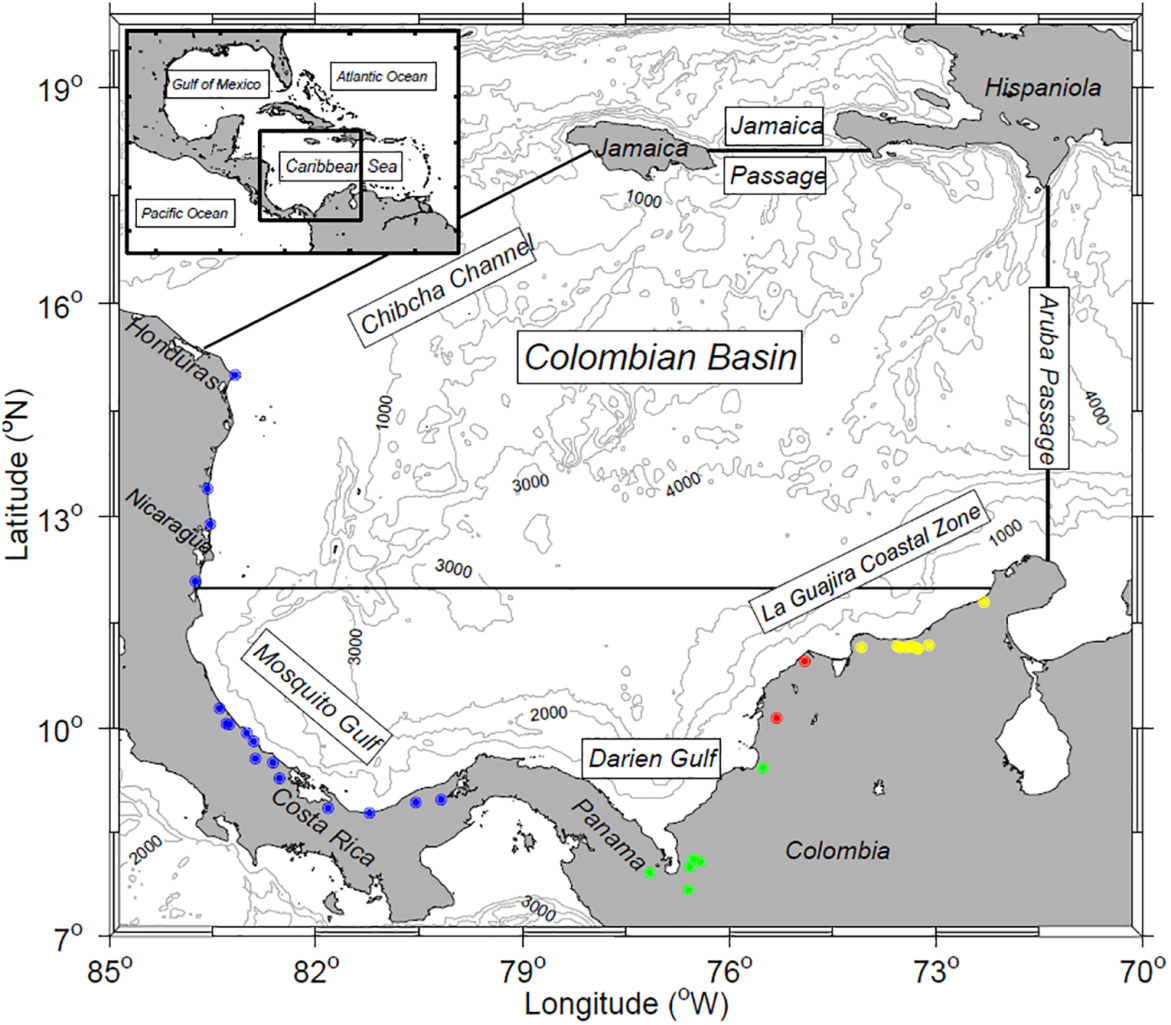
Limits of Colombian Basin with the location of the river discharges (dots). The black line at 12°N marks the boundary of the southwestern Caribbean containing the Panama-Colombia Gyre. River outflows into the zone are marked by colored dots grouped into four regions: Central American Rivers (blue), Darien Gulf Zone (green), Magdalena River Zone (red) and La Guajira Coastal Zone (yellow).

The water flux budgets strongly affect the sea surface salinity, which in combination with temperature defines the surface water masses as well as the density field, which in turn influences ocean and coastal dynamics. Salinity is also relevant to biological processes, e.g. the life cycles of a number of organisms from phytoplankton to larger predators are affected by haline concentration and its variability. Up to now, there has been no detailed investigation of the salinity regime of CSW in the Colombian Basin (Fig 1). The region south of 12°N receives the discharge of the Magdalena River [17–18], freshwater runoff from Central American rivers, and considerable precipitation over the ocean near Central America. The combined contribution of runoff and precipitation to the Colombian Basin results in strong surface salinity gradients between coastal and offshore waters [17, 19–21]. However, the variability of these salinity contrasts has not been studied in any detail.

The surface water masses entering the Caribbean Sea through the passages and channels of the Greater and Lesser Antilles are transported by the Caribbean Current but may be modified by local processes including the freshwater inflow and coastal upwelling. In this study, we use data of evaporation, precipitation, and river discharge into the Colombian Basin and water mass analysis to show that the freshwater contributions and introduction of higher salinity water to the surface layers by upwelling modify CSW along the Colombian Coast.

## Materials and methods

Precipitation over the basin was obtained from the monthly long-term mean data of the Climate Prediction Center Merged Analysis of Precipitation (http://www.esrl.noaa.gov/psd/data/gridded/data.cmap.html) with a resolution of 2.5° x 2.5° [22]. The data of Skin Temperature from the NCEP/NCAR Reanalysis with a spatial resolution of 1.86° x 1.86° (http://www.esrl.noaa.gov/psd/data/gridded/data.ncep.reanalysis.derived.surfaceflux.html) [23] and Net Latent Heat Flux from North American Regional Reanalysis, 0.25° x 0.25° (ftp://ftp.cdc.noaa.gov/Datasets/NARR/Monthlies/monolevel/apcp.mon.mean.nc) [23].

Evaporation during the common period of the three series (January 1979 to December 2012) was computed using the method described by Gill [24]:

$$E=\frac{Q_{e}}{\rho L_{v}},$$ (1)

where *E* is the evaporation rate (m s^-1^), *Q*_*e*_ is the latent heat flux (W m^-2^), *ρ* is the density of water (kg m^-3^), *L*_*v*_ *= 2*.*5008x10*^*6*^ *–(2*.*3x10*^*3*^*T)* is the latent heat of evaporation (J kg^-1^), and *T* is the surface temperature (°C).

NCEP/NCAR and NARR reanalyses are among the most dependable, have a reasonably high resolution, and are widely used by the geophysical community. As a check on data quality, our evaporation series estimated from NCEP/NCAR data at (10.625°N, 79.375°W) was compared to that in the ERA-Interim reanalysis (http://www.ecmwf.int/en/research/climate-reanalysis/era-interim) for a location in the Caribbean representative of the area south of 12° (10.75°N, 78.25°W). The two series had similar characteristics over the period of comparison 1979–2014, with high correlation *r* = 0.93 and *p-value* = 0.001, which suggests good agreement between the two series. The NCEP/NCAR estimates are used for consistency with the other analyses in this paper.

Data from 36 rivers discharging into the Colombian Basin (Table 1) were obtained from different institutions of the countries around the basin. Long-term monthly records of 17 rivers were obtained from the Colombian Hydrology, Meteorology and Environmental Institute (Instituto de Hidrología, Meteorología y Estudios Ambientales de Colombia, IDEAM, https://www.sivirtual.gov.co/memoficha-tramite/-/tramite/T209, http://institucional.ideam.gov.co/jsp/info/institucional/solicitud/solicitud.pdf); and runoff climatology of rivers from Central America were obtained from the Panama Electrical Transmission Enterprise (Empresa de Transmisión Eléctrica S.A. de Panamá, ETESA, http://www.hidromet.com.pa/hidro_historicos.php), the 21^th^ Hydrological Bulletin of the Costa Rica Electricity Institute (Instituto Costarricense de Electricidad, http://www.cfia.cr/descargas/informe1.pdf) [25] and the River Discharge Database Version 2.0 for Nicaragua (http://nelson.wisc.edu/sage/data-and-models/riverdata/index.php). Data in Table 1 were compared and completed with data of river discharges to the Colombian Basin including data from Restrepo et al. [18], Restrepo and Kjerfve [20] and Milliman and Farnsworth [26]. There are no reports of the estimation error of discharges. The data are separated into four groups in the table and figures for convenience of later reference.

**Table 1:** Annual average of the Rivers draining to the Colombian Basin.

Rivers	Country	River mouth		Period	Water discharge	
		Lat (°N)	Lon (°W)		m^3^ s^-1^	km^3^ y^-1^
Central America Rivers						
Coco	Honduras / Nicaragua	15.00	83.17	N/A	1142	36.00
Escondido	Nicaragua	12.09	83.75	N/A	824	26.00
Grande de Matagalpa	Nicaragua	12.90	83.53	N/A	920	29.00
Prinza Polka	Nicaragua	13.40	83.57	N/A	666	21.00
Mico	Nicaragua	12.07	84.54	1976–1979	40	1.26
San Juan	Nicaragua / Costa Rica	11.02	84.42	1969–1978	417	13.15
Telire	Costa Rica	9.56	82.88	N/A	172	5.42
La Estrella	Costa Rica	9.81	82.90	N/A	41	1.29
Banano	Costa Rica	9.93	83.00	N/A	25	0.79
Barbilla	Costa Rica	10.06	83.30	N/A	24	0.76
Chirripó	Costa Rica	10.05	83.26	N/A	121	3.82
Reventazón	Costa Rica	10.28	83.40	N/A	135	4.26
Sixaola	Costa Rica / Panamá	9.50	82.62	1972–2000	261	8.23
Changuinola	Panamá	9.28	82.53	1971–2009	166	5.24
Cricamola	Panamá	8.85	81.82	1975–2009	100	3.15
Calovébora	Panamá	8.78	81.22	1976–2009	63	1.99
Coclé del Norte	Panamá	8.93	80.55	1958–1986	55	1.73
Indio	Panamá	8.97	80.18	1979–2002	25	0.79
				Total	5196	163.88
Darien Gulf Zone						
Atrato	Colombia	7.72	77.15	1965–2002	2421	76.35
León	Colombia	7.28	76.39	1990–2002	15	0.47
Turbo	Colombia	8.08	76.42	1987–2002	3	0.09
Mulatos	Colombia	8.12	76.32	1977–2002	5	0.16
Currulao	Colombia	8.00	76.37	1979–2002	8	0.25
Sinú	Colombia	9.13	75.51	1965–2005	394	12.43
				Subtotal	2846	89.75
Magdalena River Zone						
Canal del Dique	Colombia	10.14	75.31	1984–1986	132	4.16
Magdalena	Colombia	10.25	74.90	1940–2004	7079	223.25
				Subtotal	7211	227.41
La Guajira Coastal Zone						
Manzanares	Colombia	11.15	74.08	1980–2005	2	0.06
Piedras	Colombia	11.17	73.56	1974–2004	5	0.16
Guachaca	Colombia	11.15	73.52	1965–2005	22	0.69
Don Diego	Colombia	11.15	73.42	1965–2004	39	1.24
Cañas	Colombia	11.13	73.26	1990–2004	9	0.28
Ancho	Colombia	11.14	73.28	1965–2005	15	0.47
Palomino	Colombia	11.16	73.34	1965–2004	25	0.79
Tapias	Colombia	11.18	73.10	1976–2004	17	0.54
Ranchería	Colombia	11.59	72.30	1990–2006	9	0.28
				Subtotal	143	4.51
				Total	15397	485.55

Lat: latitude, Lon: longitude, N/A: Not available.

High-resolution (0.25° x 0.25°) gridded ocean climatology was obtained from the National Oceanographic Data Center Web site (http://www.nodc.noaa.gov/OC5/woa13/woa13data.html), hereafter WOA13v2 data [27–28]. Among the few world databases of observed hydrography, this has the best resolution and is the most widely used. WOA13v2 hydrographic data provide objectively analyzed climatological mean fields, with greater vertical and spatial resolution than previously available [27–29].

Climatology of temperature was derived from 1 to 1 32 casts per quadrant (grid square) and salinity from 1 to 37 casts. Though salinity observations in WOA13v2 are fewer than those of temperature, it is the best available source of information on *in situ* salinity. Observations were available for between 1 and 11 months of the year in each quadrant.

Monthly mean Sea Surface Salinity (SSS) from Aquarius/SAC-D observatory was downloaded with spatial resolution of 1°x1° [30–31] from the Physical Oceanography Distributed Active Archive Center (PODAAC, http://podaac.jpl.nasa.gov/aquarius). We used Combined Active-Passive (CAPv3) Algorithm for the sea surface roughness correction to enable the retrieval of SSS, from September 2011 to April 2015 [31]. To compare the salinity data from WOA13v2 and Aquarius/SAC-D we linearly interpolated the 1-degree Aquarius data onto a 0.25-degree grid.

The climate of Colombia has been described by Mesa et al. [32]. The major dry season from December to February (DJF) is followed by a minor wet season from March to May (MAM), minor dry season (“midsummer drought”) from June to August (JJA), and major wet season from September to November (SON). We used the same seasons in our analysis. Seasonal total discharge in the rivers was calculated by regions (Table 1): Central America Rivers, Darien Gulf Zone with Atrato and Sinu as the most significant, Magdalena Rivers Zone including Dique Channel and Magdalena flow, and La Guajira Coastal Zone. As can be seen in Fig 1 and Table 1, river discharge in the Colombian Basin occurs mainly south of 12°N and can be represented by two rivers (Atrato and Magdalena) which combined contribute over 60% of the total runoff. Both rivers have long records and were used for further analysis.

For each forcing agent of SSS (evaporation, precipitation, and runoff) we have performed a seasonal fitting using Least Squares Fitting and estimations of uncertainties [33–35]:

$$F(t)=A_{0}+A_{a}\cos(\omega t-\varphi_{a})+A_{s}\cos(2\omega t-\varphi_{s})+F_{nonseason},$$ (2)

$$F_{nonseason}(t)=F(t)-F_{season}(t),$$ (3)

where *A*_*0*_ is the mean value, *A*_*α*_ and *A*_*s*_ are the annual and semiannual amplitudes for each time series; *ω = 2π/365*.*25* is the annual radian frequency; *φ*_*α*_ and *φ*_*s*_ are the phases of annual and semiannual harmonics referred to the beginning of the year; and *t* is the time. The term *F*_*nonseason*_ represents the anomalies and contains the non-seasonal variability (interannual and mesoscale). It was used to understand the interannual freshwater input into the basin, through comparisons with climatic indexes. Of the different El Niño Southern Oscillation (ENSO) indexes, the Oceanic El Niño Index appeared the most representative of the salinity forcing agents and is the one used here (ONI, http://www.cpc.ncep.noaa.gov/products/analysis_monitoring/ensostuff/ensoyears.shtml). We used the Caribbean Low Level Jet Index (CLLJ Index, Wang, 2007), defined by taking the negative of the 925hPa zonal wind anomalies in the region of 12.5°–17.5°N, 80^o^–70^o^W. Since the lower tropospheric winds in the region are easterly, the definition indicates that when the CLLJ Index is positive, the CLLJ is anomalously strong.

All time series used in this work are long enough (>25 years) to represent properly the interannual variability. To account for the effects of autocorrelation within each time series, the Effective Degrees of Freedom (*N*_*ef*_) were calculated following Trenberth [36].

To further investigate the forcing of interannual freshwater inputs in the Colombian Basin on water masses, we used information by seasons (three-monthly periods) of modeled SSS and currents near the coast during typical El Niño and La Niña years. These results correspond to outputs for the Colombian Basin over the period from 1979 to 1998 of a global run of the Parallel Oceanic Circulation Model (POCM-4C, courtesy of Dr. Robin Tokmakian from the Naval Postgraduate School, Monterey, CA, USA). The spatial and temporal resolution of the model was 0.25°x0.25° and one month, respectively. The POCM model [37–38] reproduces properly in qualitative terms the seasonal and interannual variability of large scale circulation [39–40].

## Results and interpretation

### Fresh water inputs

**Fig 2:**
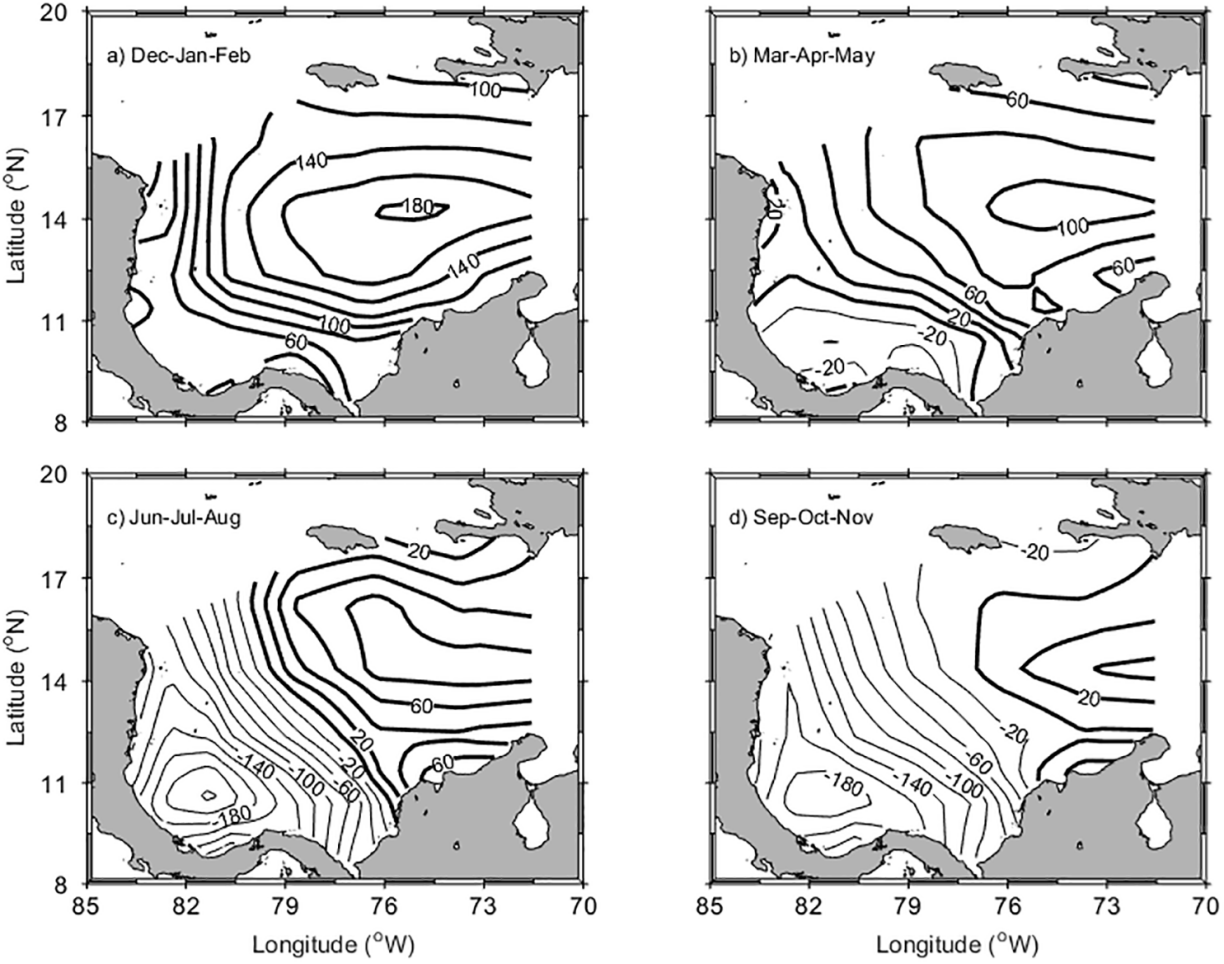
Evaporation minus precipitation (E-P) over the Colombian Basin by seasons. Heavy contours indicate positive values, where evaporation exceeds precipitation (concentration conditions) and light contours indicate negative values (dilution conditions).

The climatological seasonal maps of evaporation minus precipitation (E-P) (mm month^-1^) are shown in Fig 2. From the central Colombian Basin toward the Mosquito and Darien Gulfs, (Fig 1) there is a gradient of E-P during the whole year. The high E-P values in the eastern, central half of the basin coincide with the core of the CLLJ. During the main dry season (DJF) evaporation dominates all over the basin from a maximum 180 mm month^-1^ in the central basin to a minimum of 40 mm month^-1^ in the Mosquito and Darien Gulfs. In MAM precipitation exceeds evaporation in the Mosquito Gulf. E-P values continue decreasing during the rest of the year (JJA and SON), with dilution in the southwest and concentration in the northeast. During the “midsummer drought” (JJA) the lowest values of E-P occur close to Central America, and during the main wet season (SON) the area of dilution extends as far as 76°W. The spatial pattern of dilution and concentration basins during the second half of the year reflects the Walker-type convection cell and the divergence of winds over the Colombian Basin [41–43].

**Fig 3:**
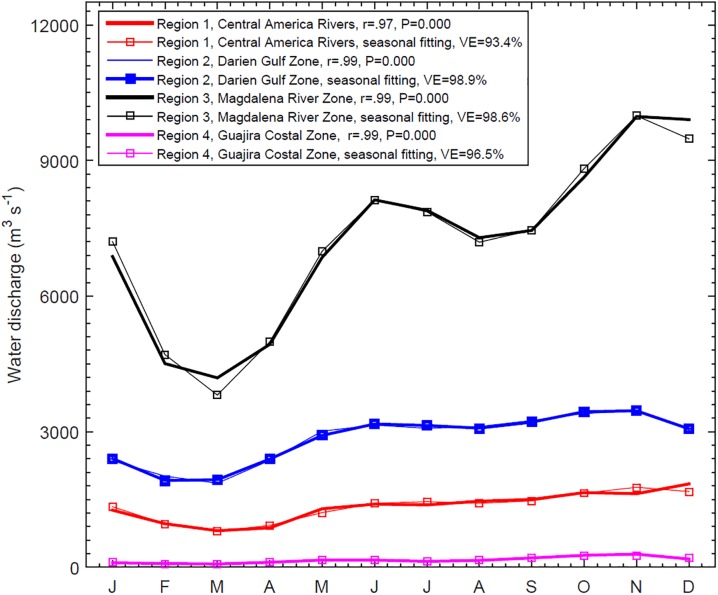
Seasonal cycle of river discharge into the Colombian Basin. The regions as shown in Table 1 and Fig 1. The thin lines represents the seasonal fitting, and the respective percentage of explained variance is included.

The total annual mean river discharge into the Colombian Basin is estimated at 15397 m^3^ s^-1^ (Table 1). This is equivalent to about ~50% of the annual mean discharge of the Orinoco River (31000 m^3^ s^-1^) [7] into the eastern Caribbean. Over 60% of total water discharge directly into the Colombian basin arises from only two rivers, the Magdalena (7100 m^3^ s^-1^) and the Atrato (2400 m^3^ s^-1^). Rivers north of 12°N make a smaller contribution to the overall budgets. The climatological monthly mean of rivers draining to the Colombian Basin has a strongly seasonal signal, as can be seen in Fig 3 where the climatological values (thick lines) are very close to the seasonal fit (thin lines). Seasonal fitting explains from 93% to 99% of the climatological variance. Water discharges have a bimodal seasonal cycle (similar annual and semiannual amplitudes) with maximums in October–November and May–June, and minimums in February–March and July–August, consistent with the wet and dry seasons. The sum of all river runoff in the Colombian Basin has the minimum (maximum) runoff values in March (November), e.g., Magdalena River contributes around 10000 m^3^ s^-1^ in November and diminishes to 4000 m^3^ s^-1^ in March.

**Fig 4:**
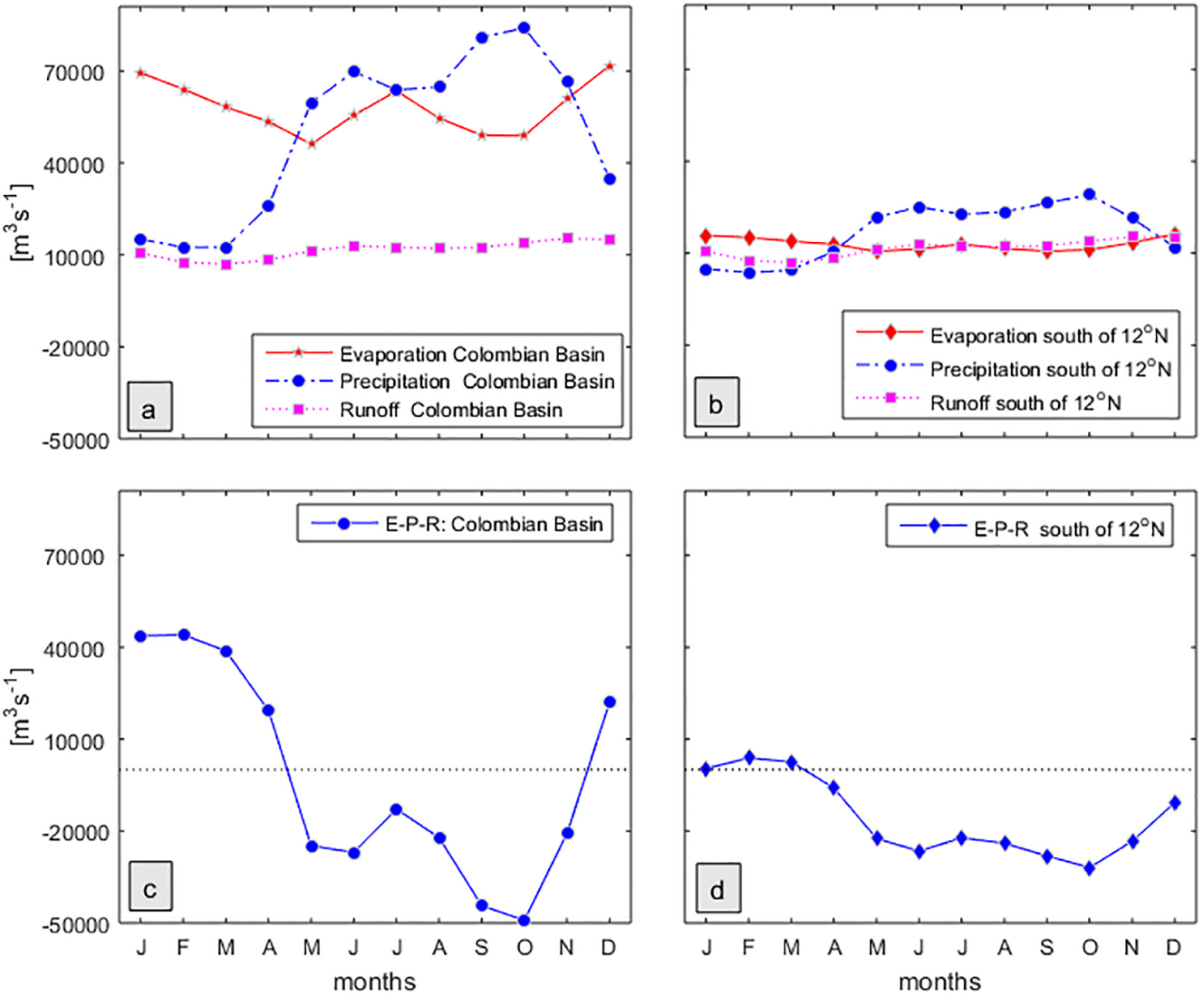
Monthly means of evaporation, precipitation and runoff. (a, b) and the resulting budget (c, d) over the entire Colombian Basin (a, c) and the area south of 12°N (b, d).

Taking into account the E-P and runoff shown in Figs 2 and 3, most of the surface water dilution in the Colombian Basin occurs south of 12°N. Climatological monthly means of evaporation (E), precipitation (P) and runoff (R), as well as the resulting budget between them, E-(P+R), are depicted in Fig 4A and 4C for the entire Colombian Basin and for the area south of 12°N Fig 4B, 4A and 4D, respectively.

Overall, in the Colombian Basin, precipitation and evaporation are up to 5 times higher than runoff (Fig 4A) while south of 12°N all are more similar in magnitude (Fig 4B). From May to November, the Colombian Basin is a dilution basin while from December to April it is a concentration basin (Fig 4C). The region south of 12°N suffers net dilution from April to December and only weak concentration in the remaining months (Fig 4D). Total evaporation in the Colombian basin is 57965 m^3^ s^-1^ and total precipitation 49184 m^3^ s^-1^.

The net evaporation for the Colombian Basin E-(P+R) is -6616 m^3^ s^-1^, an order of magnitude smaller than E, P or R. But the freshwater budget for the area south of 12°N is determined by evaporation of 12959 m^3^ s^-1^, precipitation of 17105 m^3^ s^-1^ and runoff at 11845 m^3^ s^-1^, and so the net balance of E-(P+R) = -15991 m^3^ s^-1^. Given that, the sum and its constituent terms all have the same order of magnitude; this sub-region appears to be a zone of net dilution. However, relative errors in each of the terms may be large, and more observations are needed to confirm this result.

### Surface water properties

**Fig 5:**
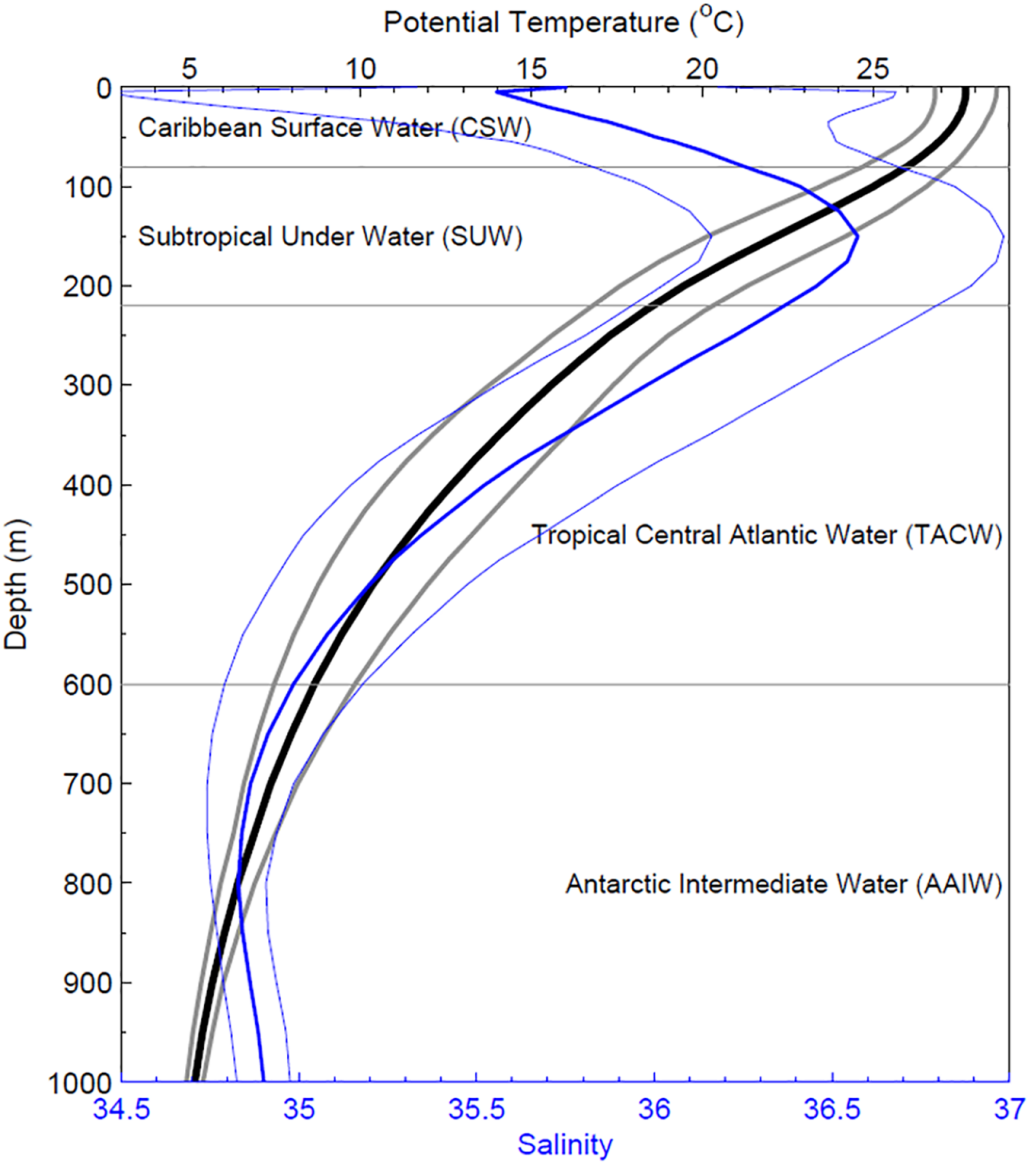
Mean vertical profiles of temperature (thick black) and salinity (thin blue) based on data from WOA13v2. The lighter curves indicate range of the data, while horizontal lines indicate water mass limits.

Using the WOA13v2 data in the Colombian Basin, the main Caribbean water masses reported in previous works [5, 15, 44–46] can be identified. The mean potential temperature and salinity profiles as a function of depth, averaged over the whole Colombian Basin with their respective minimums and maximums (Fig 5), show a wide variation of the salinity of the CSW. Centered at 150 m, the Subtropical Underwater (SUW) is seen as a typical salinity maximum of 36.5 above the deeper Tropical Atlantic Central Water (TACW) and Antarctic Intermediate Water (AAIW) layers.

**Fig 6:**
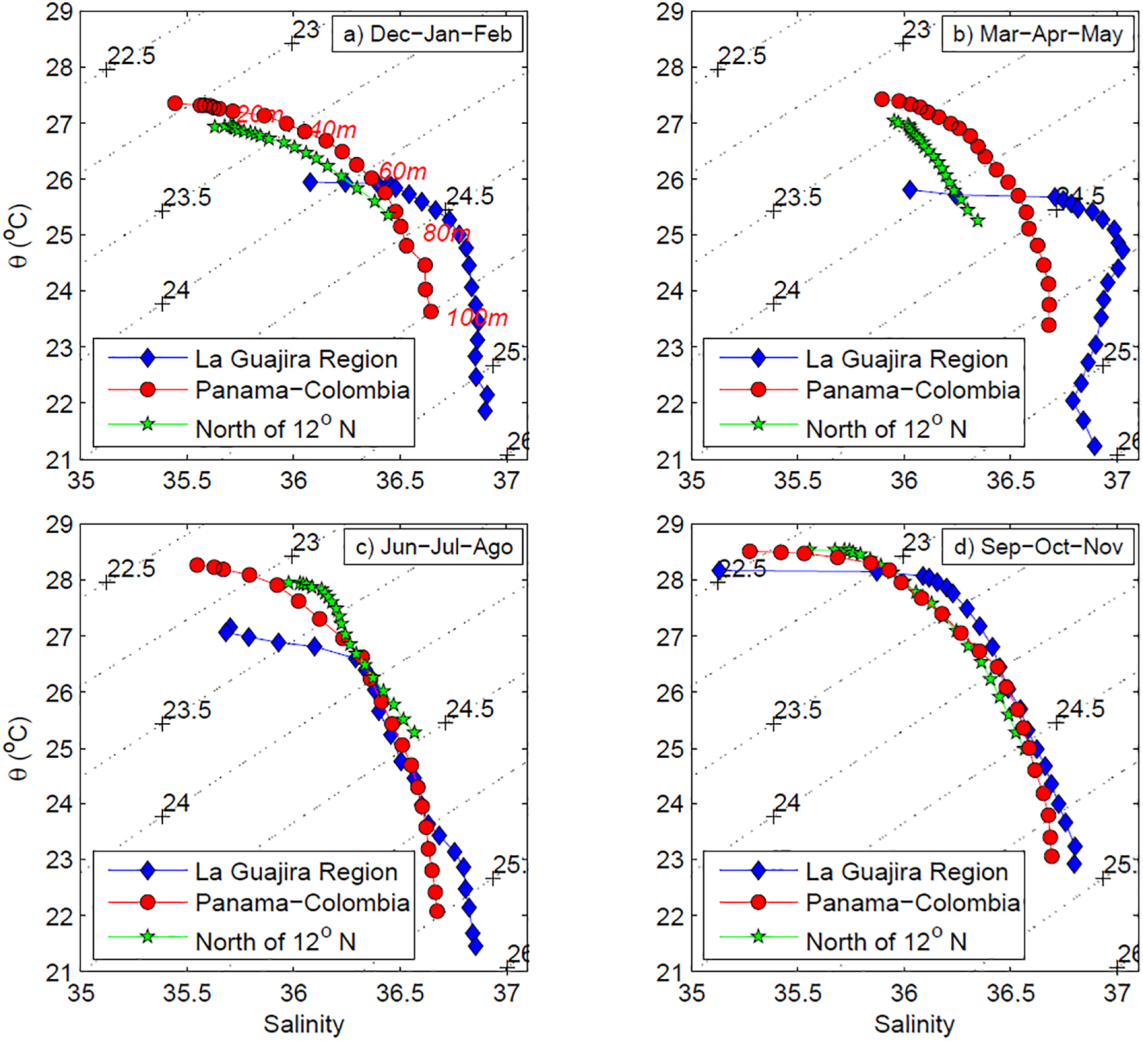
Seasonal θ-S diagrams in the Colombian Basin for the upper 100 m layer. Data from WOA13 hydrographic climatologic data, averaged by depths in three areas. (a) North of 12°N, (b) La Guajira (South of 12°N and East 74°W) and (c) Panamá-Colombia (South of 12°N and West 74°W).

The spatial and seasonal changes can be seen in detail in the θ-S diagrams of surface waters down to 100 m depth (Fig 6) averaged by depths and regions as follows: north of 12°N; south of 12°N and east of 74°W (La Guajira upwelling zone); and south of 12°N and west of 74°W (the intense precipitation area of the Panama-Colombia Gyre). Surface waters are within the intervals reported for CSW (temperature between 25 and 29°C; salinity higher than 35.1) but, in the area of the Panama-Colombia region south of 12°N, waters above 30 m are fresher and warmer all year than those north of 12°N. Meanwhile, in La Guajira region, surface waters are significantly colder than in the other regions from December through May because of strong upwelling [47–48]. During these months, a thin (~10 m) low salinity surface layer is present, but waters between 10 and 100 m are significantly saltier than in the other regions (also because of the upwelling). In JJA, surface water in la Guajira is colder but with the same salinity as the Panama–Colombia region (lower than the Central Caribbean). During SON, when upwelling is weaker, water masses of the three regions become similar, except for the shallowest surface waters (above 5 m) of Panama-Colombia and La Guajira whose salinity remains low.

**Fig 7:**
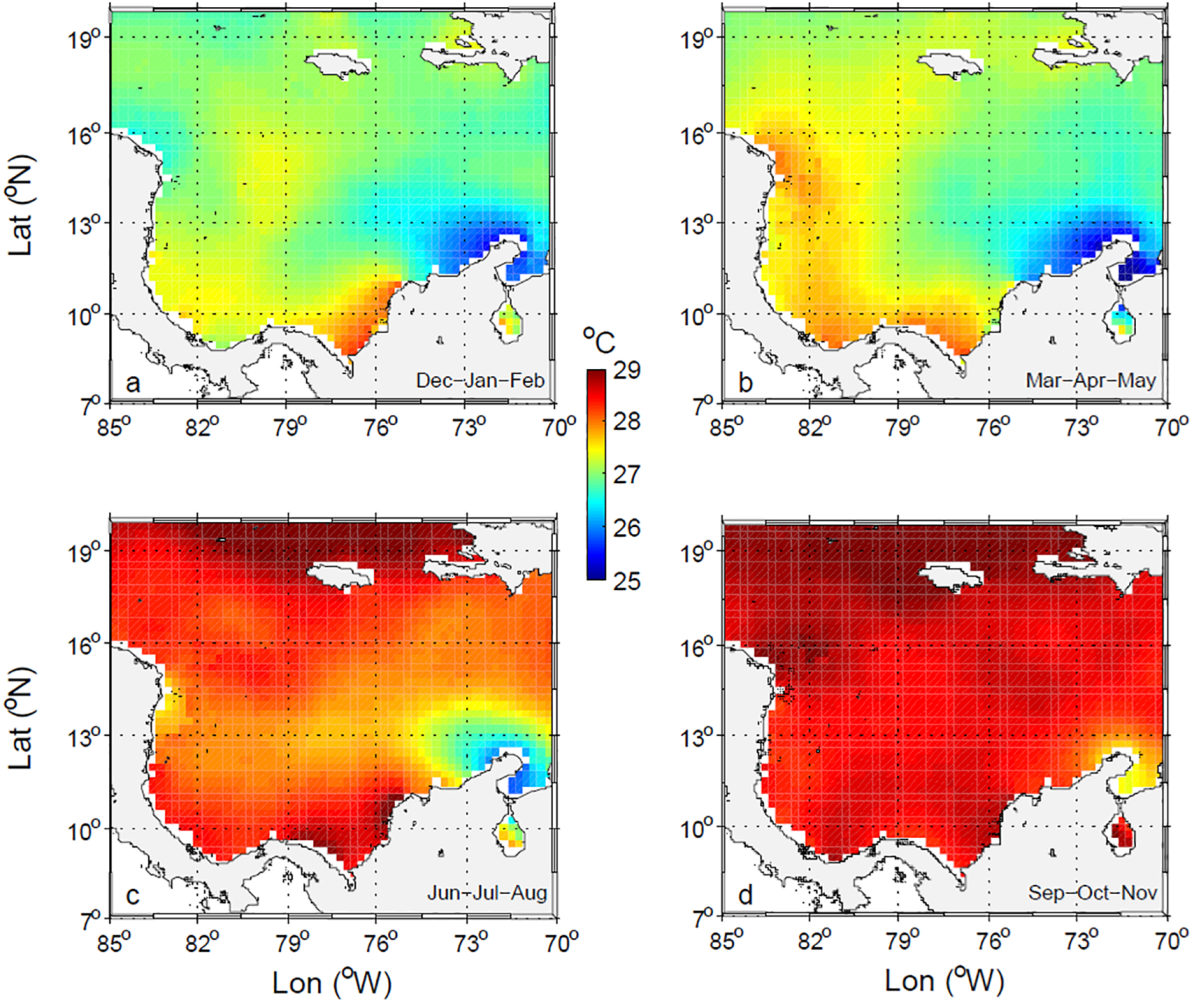
Seasonal variability of sea surface temperature [°C] from WOA13 climatological data. (a) DJF, (b) MAM, (c) JJA, and (d) SON.

The seasonal distributions of WOA13v2 SST (Fig 7) show the strong surface cooling produced by the upwelling in La Guajira Coastal Zone particularly during DJF and MAM. During JJA and SON, although La Guajira region remains locally cooler, surface heating is evident throughout the basin, including the regions of anomalously high temperatures in the Darien and Mosquitos Gulfs (see Fig 1). These anomalies were reported and correlated with surface forcing by Ruiz-Ochoa et al. [41] based on Pathfinder satellite SST. The strong similarity of the temporal and spatial patterns from WOA13v2 and Pathfinder SST confirms the capability of the former to characterize the climatology of hydrography in the basin, although there are fewer salinity than temperature observations in the database.

**Fig 8:**
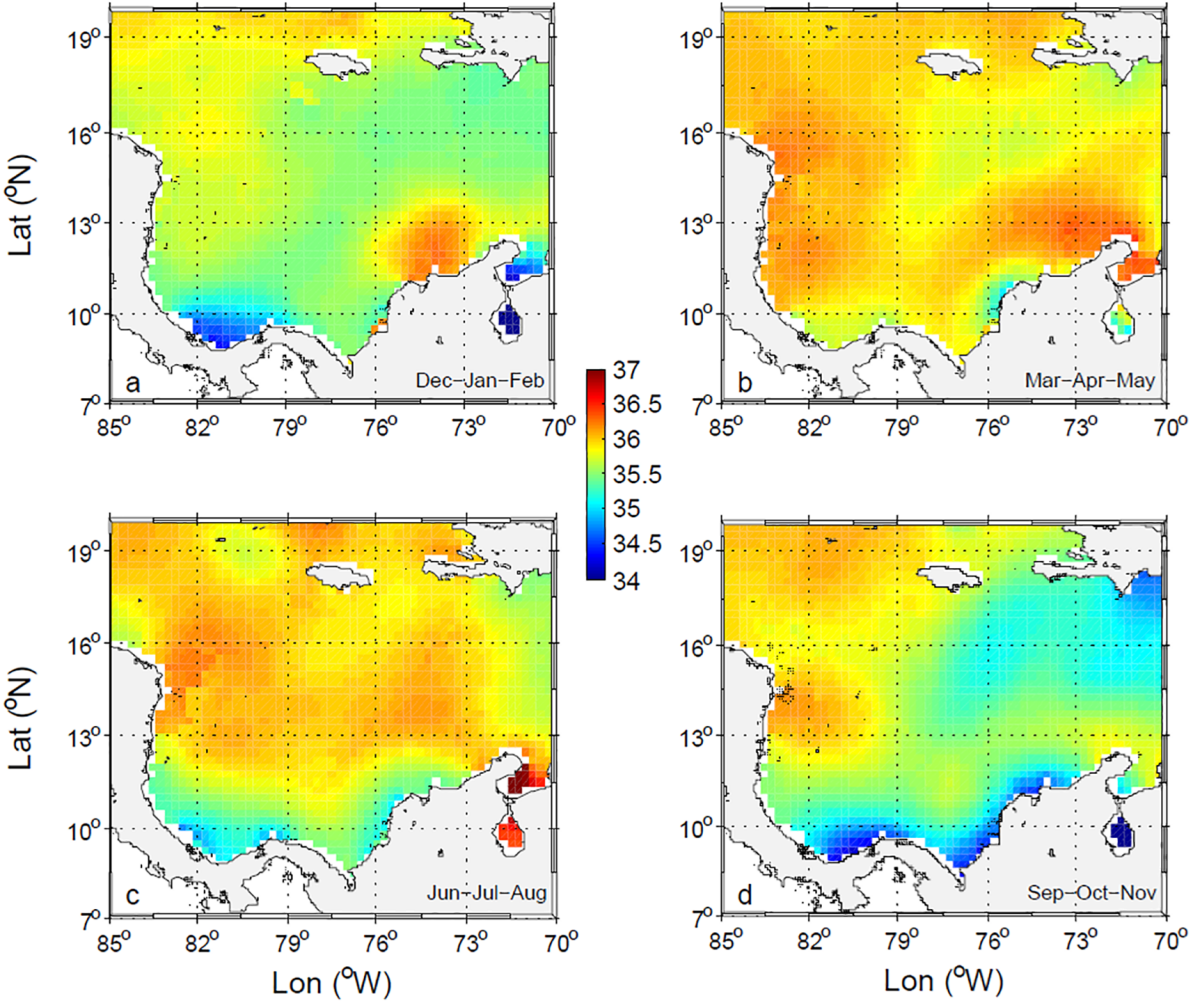
Seasonal variability of sea surface salinity from WOA13 climatological data. (a) DJF, (b) MAM, (c) JJA, and (d) SON.

On the basis of WOA13v2 salinity surface quarterlies (Fig 8), the central basin salinities are generally highest in MAM and JJA, and lowest in SON, the period of most rain and advection of waters diluted by the Orinoco from the Atlantic [5, 11]. The cooler waters of La Guajira region are also generally saltier than the rest of the basin. In contrast with the saltier waters off La Guajira, fresher waters of salinities as low as 34.2 occur in the Darien and Mosquito Gulfs, coincident with higher temperatures. These low salinity waters extends eastwards along the Colombian coast in JJA reaching their maximum extension in SON, when precipitation and runoff are maximum. The eastward extension of low salinity waters is minimal in MAM.

The spatial distributions of sea surface salinity and temperature suggest that in the Colombian Basin the CSW is diluted south of 12°N, where it acquires lower salinities and higher temperatures. Waters in the basin are more homogenous in MAM, after the main dry season, when they are saltier, and in SON, the main rainy season, when they are fresher. Off La Guajira, the colder and saltier water associated with the upwelling of SUW in DJF and MAM becomes fresher and warmer in SON because of the advection of waters from the west. From DJF through MAM, the intensity of the CLLJ and wind stress curl along the La Guajira Coast is most favorable to upwelling [41].

The recent availability of remotely sensed sea surface salinity from the Aquarius mission provides an independent check on the salinity field for the basin. Although Aquarius data are currently available only from September 2011 to April 2015 and are known to be unreliable close to shore, they still provide useful information on the region. We calculated seasonal means of these data using only pixels flagged as valid and free from land contamination. The seasonal composites over this period (2011–2015) showed higher salinity values (between 0.25 and 0.5) but a similar seasonal pattern to the WOA13v2 data (*r* = 0.72, *p-value* = 0.001) despite the different record lengths and the low spatial resolution of Aquarius.

### Interannual variability of freshwater inputs and water masses

The seasonal cycles explain 61% of the total variance of E, 80% of P, and 53% and 30% of river outflow (R) from Magdalena and Atrato rivers, respectively. Fitting errors of amplitudes and phases are low. Monthly means of Magdalena runoff, *M*_*nonseason*_, from 1950 to 2004, 648 observations, correlate with the ONI Index with an *r =* -0.52, *p-value*<10^−10^, *N*_*ef*_ = 168 and a lag of one month. This suggests that during El Niño (La Niña) events, the Colombian Basin receives less (more) runoff. Furthermore, the Magdalena anomalies of discharge (*M*_*nonseason*_) are correlated with the Niño1+2 Index, which represents the SST in the extreme Eastern Tropical Pacific, with *r* = -0.64, *p-value*<10^−11^, *N*_*ef*_ = 172 and a lag of one month. Similar values of correlation coefficient and statistical significance were found between the Atrato river outflow and ONI index.

**Fig 9:**
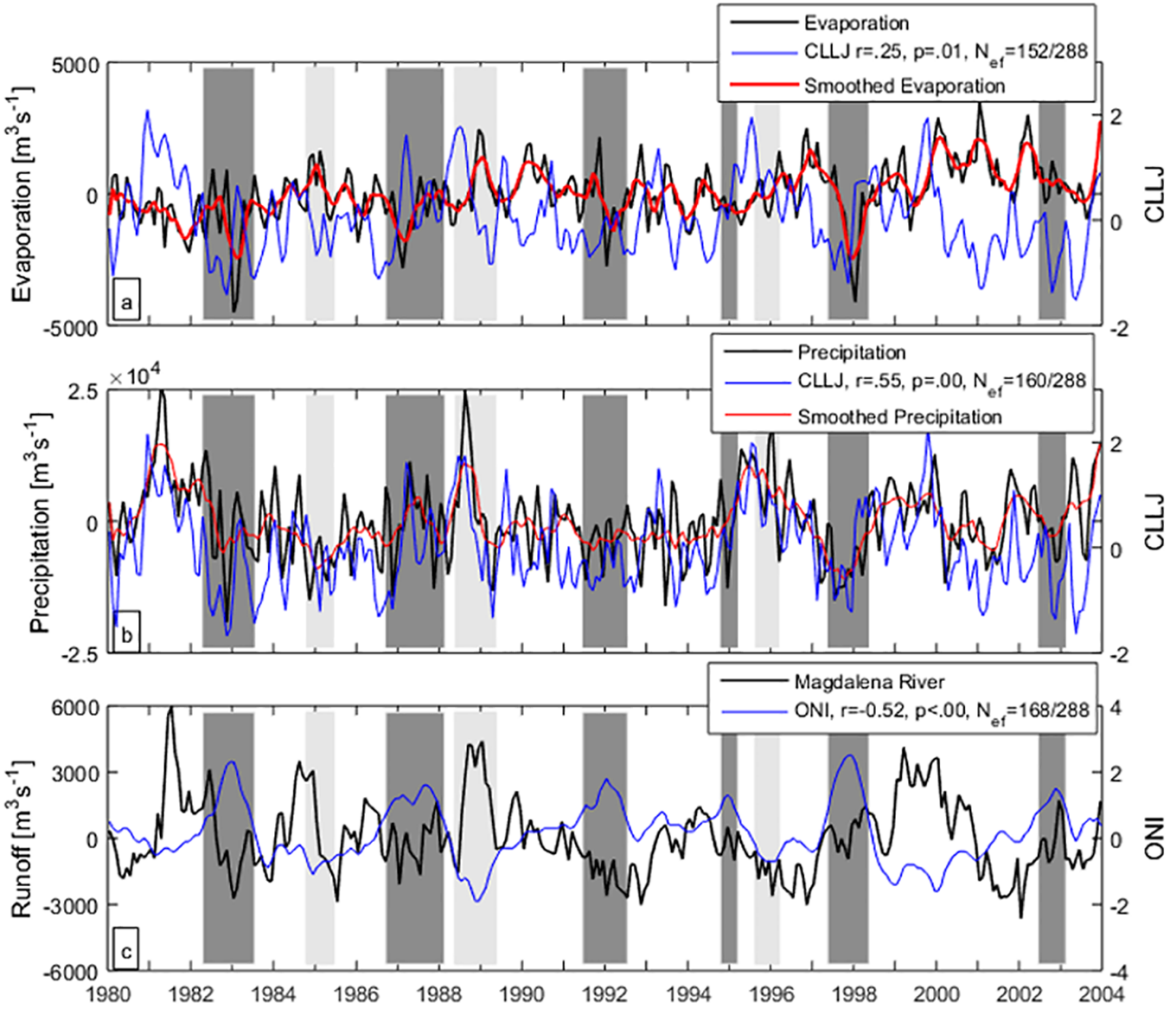
Anomalies of evaporation and precipitation south of 12°N and water discharge of Magdalena River. In dotted lines their best-correlated index (CLLJ Index and ONI, respectively). El Niño (dark gray) and La Niña (light gray) periods according to ONI are shaded. Red lines show 5-month moving averages of E and P.

Monthly means of *P*_*nonseason*_ from 1980 to 2004, 288 observations, correlate well with the CLLJ, *r* = 0.55, *p-value*< 10^−10^, *N*_*ef*_ = 160 with zero lag. Finally, *E*_*nonseason*_ from 1979 to 2008, 348 observations, correlates with the CLLJ with *r* = -0.31, *p-value*< 10^−7^, *N*_*ef*_ = 271 with no lag. Time series of anomalies of surface forcing of freshwater balance south of 12°N during the common period between 1980 and 2004 are shown in Fig 9, with El Niño and La Niña periods (notice that *r*, *p-value*, and *N*_*ef*_ changes with time series length). For each time series Fig 9 also shows the relation with the interannual variability through the CLLJ Index (Fig 9A and 9B), and ONI (Fig 9C). The sum of P+R in Fig 9B and 9C is one order of magnitude higher than E. The figure shows that E and R decrease during El Niño, and increase during La Niña periods. The pattern for precipitation in relation with El Niño or La Niña is not clear, whenever it is not well correlated with ENSO.

**Fig 10:**
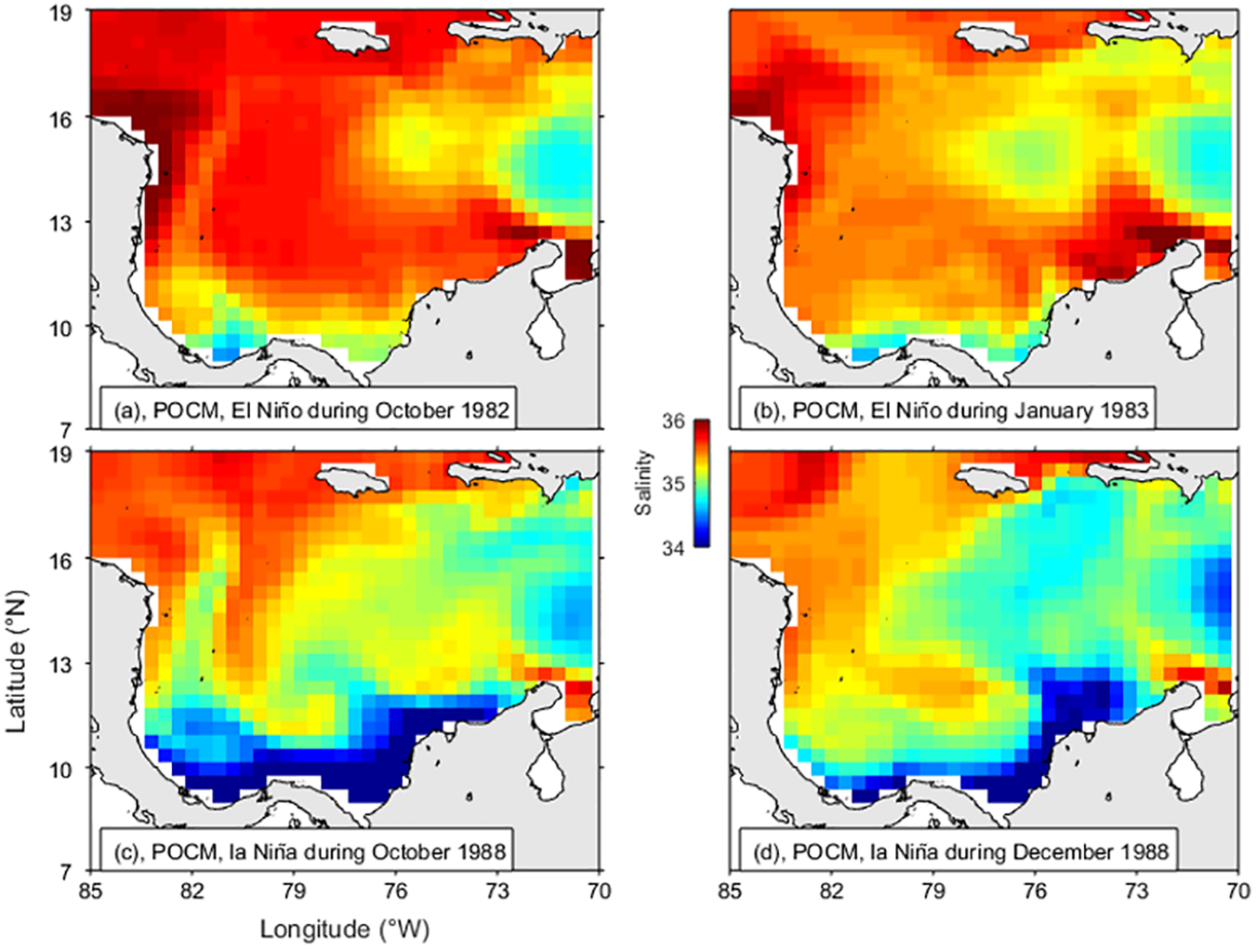
Parallel Oceanic Circulation Model (POCM-4C) outputs of sea surface salinity (12.5 m). (a) Wet season of El Niño 1982–83, (b) Dry season of El Niño 1982–83, (c) Wet season of La Niña 1988–89, and (d) Dry season of La Niña 1988–89.

**Fig 11:**
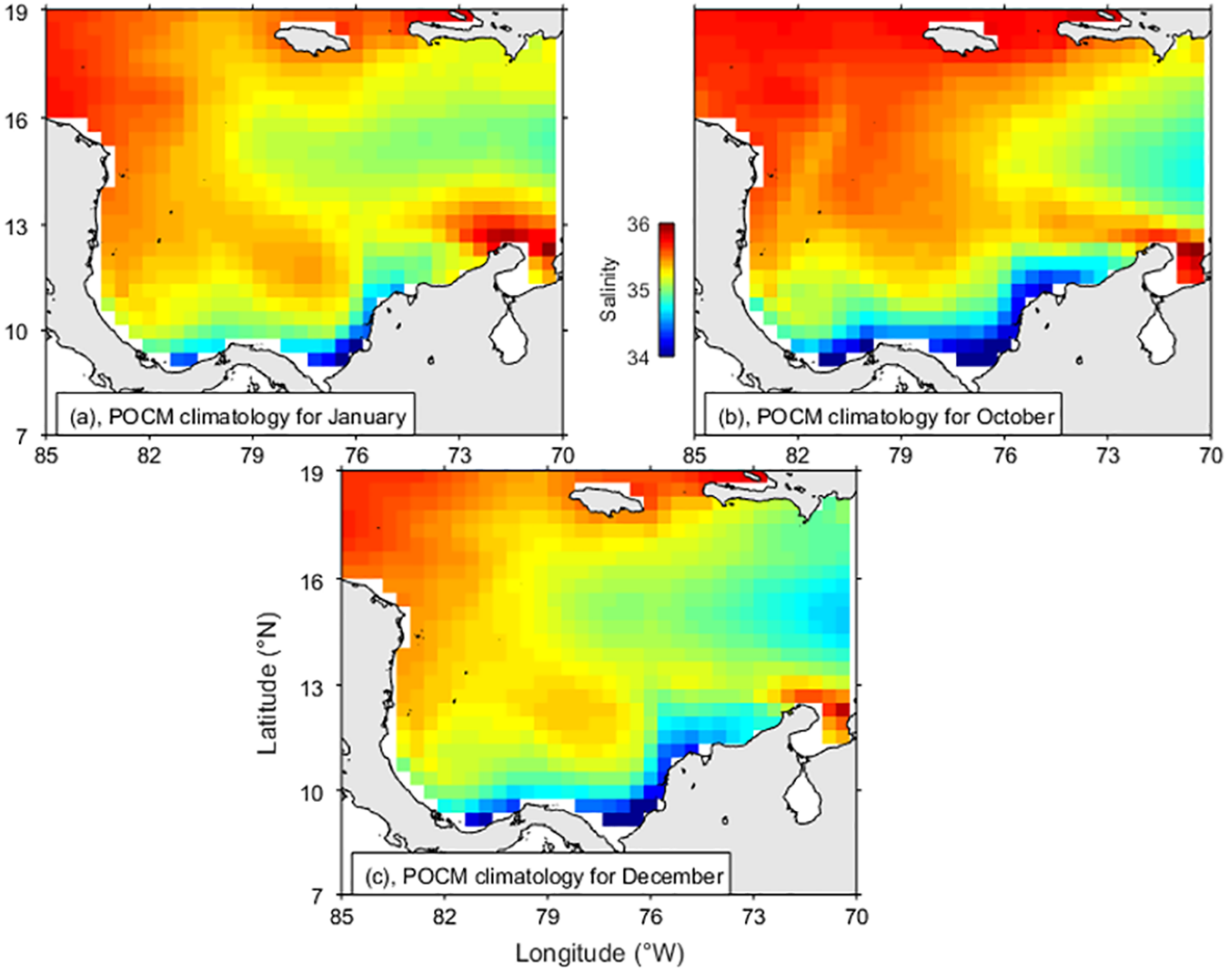
Parallel Oceanic Circulation Model (POCM-4C) outputs of sea surface salinity (12.5 m). (a) January climatology, (b) October climatology, and (c) December climatology.

Output from the POCM-4C was used to test qualitatively if ENSO phases affect the river discharges sufficiently to modify the coastal surface water masses. Here Caribbean SSS maps were extracted from the global run over the period 1980 to 1998 as explained in the Methods section. Results for contrasting years show that during El Niño of 1983 (Fig 10) the low salinity coastal water in the Panama-Colombia Gyre was less extended and the high salinity off La Guajira was more extended than in the climatology of POCM, for both dry and wet seasons (Fig 11). Conversely, during La Niña of 1988 (Fig 10), the low salinity coastal water was more extended than in the climatology of POCM (Fig 11) and extended as far as La Guajira. Although the model captures the seasonality only qualitatively, it represents the general oceanography of the Colombian Basin and the spatial distribution of the water masses. Therefore, it serves as a first approximation to their interannual behaviour and represents the ENSO variability reasonably well.

## Discussion and conclusions

The Caribbean Sea is generally considered a concentration basin where evaporation exceeds precipitation [1–4]. Remotely sensed SSS and numerical modelling indicates that, on an interannual basis, the salinity of water entering the Caribbean through the Lesser Antilles can vary by 0.5 psu [8]). This results from variability of the Amazon plume caused by changes in discharge rates and in the ocean and atmospheric forcing that govern its extent. Thus interannual anomalies of salinity unrelated to local conditions propagate westward through the central Caribbean and on into the Gulf Stream system. On the other hand, our results show that variability in the southwestern Caribbean is related to the local conditions. Unlike the rest of the Caribbean, the Colombian Basin is not a concentration basin, and south of 12°N it is a strong dilution basin in which the contribution of runoff to the water balance is as important as evaporation and precipitation (Fig 5D).

The net dilution in the southwest and upwelling in the northeast of the southern Colombian Basin modifies CSW, which shows wide variation of its salinity, as noted by Andrade and Barton [49]. Furthermore, Corredor [47], reported alternation of surface waters off La Guajira Peninsula between properties of the Panama-Colombia Countercurrent and wind-induced coastal upwelling. *In situ* observations by Andrade and Barton [48] and simulations by Lonin et al. [50] found that warm and low salinity waters from the Panama-Colombia Gyre block the southwestward extension of La Guajira coastal upwelling that normally reaches 10.5° N. During upwelling, we found a thin surface layer (0–10 m) of low-salinity water with very salty water between 10 and 100 m. Presumably when upwelling is weaker off La Guajira, the western water mass penetrates further to the east.

Interannual variability of the evaporation and precipitation in the south of the Colombia Basin correlates better with the local forcing of the CLLJ Index than with ENSO (ONI). This permanent low-level wind jet over the basin [51–55] enhances evaporation, transports humidity and forms strong convection over topography in Central America. Conversely, runoff is better correlated with ENSO (ONI) because it originates principally in continental Colombia where, during El Niño events, precipitation and runoff decrease, while La Niña events have the contrary effects [19, 32, 56–60]. Even though the annual cycles of precipitation and runoff are related, the deseasonalized time series are poorly correlated (*r* = 0.19, *p-value* < 0.03, *N*_*eff*_ = 129 over 288 observations), because of the isolation of the catchment area of the Colombian rivers in the northern Andes from the Caribbean. Although both spatial patterns of precipitation over the Colombian mainland and their temporal variability are complicated, it has been recognized that ENSO is the greatest single cause of interannual variability within the region (e.g. Poveda et al. [57]) as indicated here by the significant correlation between the ONI and Magdalena River outflow.

Amador [54] reported that during El Niño (warm) phases, stronger than normal CLLJ winds occur, while during La Niña (cool) phases the CLLJ is weaker, in both cases especially during summer. A stronger CLLJ was found to be associated with greater than normal precipitation in the western Caribbean and weaker precipitation in the central Caribbean, while a weaker CLLJ produced the opposite. Our results for that portion of the Colombian basin south of 12°N show that precipitation is indeed positively correlated with the CLLJ Index, as expected. However, the precipitation anomalies were not well correlated with ENSO. We found that during the El Niño dry season (boreal summer) lower runoff results in less dilution of CSW in the Panama-Colombia Gyre and greater presence of upwelling waters (enhanced also by stronger winds of the CLLJ). In contrast, during the wet season of La Niña, runoff extends dilution beyond its normal range to reach as far as La Guajira Coastal Zone.

The interplay between warm and low salinity waters from the Panama-Colombia Gyre and cold and high salinity waters from La Guajira is related to the zonal wind circulation that plays a role in the Caribbean–Pacific connection [41–43]. The CLLJ is the surface component of the cell with upward convective processes at the Caribbean side of Central America, and low-level divergence and downward drying over La Guajira. According to Andrade and Barton [42] and Hidalgo et al. [43], the convergent zone is connected with another cell in the west, with the sinking of dry air over the Eastern Tropical Pacific, where the drying favors the southward displacement of the Inter Tropical Convergence Zone (ITCZ). This could be the link between ENSO and CLLJ that produces the seasonal and interannual patterns of water masses observed here.

La Guajira upwelling is part of the South Caribbean Upwelling System [61]. It is characterized by strong physical forcing [41, 48, 62–63], strong mixing of subsurface water with surface water, but low chlorophyll concentration [63–64]. Different explanations reported for the low productivity in La Guajira include oligotrophic source waters [16], the upwelling of Panama-Colombia undercurrent water [63], a greater depth of the SUW in front of La Guajira than off Venezuela, or reduced efficiency of wind energy transfer [64]. Our findings indicate that from June to November, surface waters off La Guajira (see Figs 6 and 8) are similar to the western diluted CSW; especially during La Niña years (see Fig 10). This low salinity water, originating in the southwest of the basin, favors a strong surface stratification that inhibits vertical mixing and enhances surface heating and thus, plays a role in the low productivity off La Guajira.

Based on observed surface temperature, salinity and runoff, a first evaluation of the seasonal surface water balance of the Colombian Basin has been made. Results demonstrate that south of 12°N in the Colombian Basin, the surface water balance indicates a net dilution, responsible for producing a strong dilution of CSW. This water reaches the La Guajira region from June to November, where it dilutes saltier water produced by local upwelling.

At interannual scale, precipitation and evaporation are related to variability of the CLLJ, while runoff is related to ENSO. In turn, the CLLJ can change in relation to ENSO. Since south of 12°N runoff has the same order of magnitude as precipitation, the result is an anomalous decrease in the area of dilution and persistence of saltier water off La Guajira during the dry season of El Niño. In contrast, during the wet season of La Niña, the area of dilution extends beyond its normal range to reach La Guajira Coastal Zone. The intensified seasonal invasion of La Guajira upwelling by warm and freshwater from the Panama-Colombia Gyre during La Niña events favors surface stratification and can contribute to the low productivity of this upwelling system.

## Supporting information

S1 Appendix List of acronyms. (DOCX)

## References

[1] Etter PC, Lamb PJ, Portis DH. Heat and freshwater budgets of the Caribbean Sea with revised estimates for the Central American Seas. J Phys Oceanogr. 1987;17: 1232–1248.

[2] Yoo JM, Carton JA. Annual and interannual variation of the fresh-water budget in the Tropical Atlantic-Ocean and the Caribbean Sea. J Phys Oceanogr. 1990;20(6): 831–845. doi: 10.1175/1520-0485(1990)020<0831:AAIVOT>2.0.CO;2

[3] Tomczak M, Godfrey J. Regional Oceanography: An introduction. 2nd ed New Delhi: Daya Publishing House; 2003.

[4] Schmidt MW, Spero HJ, Lea DW. Links between salinity variation in the Caribbean and North Atlantic thermohaline circulation. Nature. 2004;428: 160–163. doi: 10.1038/nature02346 15014495 10.1038/nature02346

[5] Kelly PS, Lwiza KMM, Cowen RK, Goni GJ. Low salinity pools at Barbados, West Indies: The origin, frequency, and variability. J Geophys Res Oceans. 2000;105(C8): 19699–19708. doi: 10.1029/1999JC900328

[6] Morrison JM, Smith OP. Geostrophic transport variability along the Aves Ridge in the eastern Caribbean Sea during 1985–1986. J Geophys Res Oceans. 1990;95(C1): 699–710. doi: 10.1029/JC095iC01p00699

[7] Hu C, Montgomery ET, Schmitt RW, Muller-Karger FE. The dispersal of the Amazon and Orinoco River water in the tropical Atlantic and Caribbean Sea: Observation from space and S–PALACE floats. Deep Sea Res Part 2 Top Stud Oceanogr. 2004;51: 1151–1171. doi: 10.1016/j.dsr2.2004.04.001

[8] Grodsky SA, Johnson BK, Carton JA, Bryan FO. Interannual Caribbean salinity in satellite data and model simulations. J Geophys Res Oceans. 2015;120: 1375–1387. doi: 10.1002/2014JC010625

[9] Fratantoni DM. North Atlantic surface circulation during the 1990’s observed with satellite-tracked drifters. J Geophys Res Oceans. 2001;106(C10): 22,067–22,093. doi: 10.1029/2000JC000730

[10] Richardson PL. Caribbean Current and eddies as observed by surface drifters. Deep Sea Res Part 2 Top Stud Oceanogr. 2005;52: 429–463. doi: 10.1016/j.dsr2.2004.11.001

[11] Chérubin LM, Richardson PL. Caribbean Current variability and the influence of the Amazon and Orinoco freshwater plumes. Deep Sea Res Part 1 Oceanogr Res Pap. 2007; 54: 1451–1473. doi: 10.1016/j.dsr.2007.04.021

[12] Jouanno J, Sheinbaum J, Barnier B, Molines J-M, Debreu L, Lemarié F. The mesoscale variability in the Caribbean Sea. Part I: Simulations and characteristics with an embedded model. Ocean Model. 2008;23: 82–101. doi: 10.1016/J.OCEMOD.2008.04.002

[13] Centurioni LR, Niiler PP. On the surface currents of the Caribbean Sea. Geophys Res Lett. 2003;30(6): 1279 doi: 10.1029/2002GL016231

[14] Alvera-Azcárate A, Barth A, Weisberg RH. The surface circulation of the Caribbean Sea and the Gulf of Mexico as inferred from satellite altimetry. J Phys Oceanogr. 2009;39: 640–657. doi: https://doi.org/10.1175/2008JPO3765.1

[15] Mooers CNK, Maul GA. Intra-Americas Sea circulation In: Robinson AR, Brink KH, editors. The Sea, vol. 11: Regional Studies and Syntheses. New York: John Wiley; 1998 pp. 183–208.

[16] Andrade CA, Barton ED, Mooers CNK. Evidence for an eastward flow along the Central and South American Caribbean Coast. J Geophys Res Oceans. 2003;108(C6): 3185–3202. doi: 10.1029/2002JC001549

[17] Restrepo JD, Zapata P, Díaz J, Garzón-Ferreira J, García C. Fluvial fluxes into the Caribbean Sea and their impact on coastal ecosystems: The Magdalena River, Colombia. Global Planet. Change. 2006;50(1–2): 33–49. doi: 10.1016/j.gloplacha.2005.09.002

[18] Restrepo JC, Ortiz JC, Pierini J, Schrottke K, Maza M, Otero L, et al Freshwater discharge into the Caribbean Sea from the rivers of Northwestern South America (Colombia): Magnitude, variability and recent changes. J Hydrol. 2014;509: 266–281. doi: 10.1016/j.jhydrol.2013.11.045

[19] Restrepo JD, Kjerfve B. Magdalena River: Interannual variability (1975–1995) and revised water discharge and sediment load estimates. J Hydrol. 2000;235(1–2): 137–149. doi: 10.1016/S0022-1694(00)00269-9

[20] Restrepo JD, Kjerfve B. The Pacific and Caribbean Rivers of Colombia: Water discharge, sediment transport and dissolved loads In: Lacerda L, Santelli R, Duursma E, Abrao J, editors. Environmental geochemistry in tropical and subtropical environments. Berlin: Springer Verlag; 2004 pp. 169–187.

[21] Cañón M, Santamaría-del-Ángel E. Influencia de la pluma del río Magdalena en el Caribe colombiano. Bol Cient CIOH. 2003;21: 66–84.

[22] Xie P, Arkin PA. Global precipitation: A 17-year monthly analysis based on gauge observations, satellite estimates, and numerical model outputs. Bull Amer Meteor Soc. 1997;78(11): 2539–2558. doi: 10.1175/1520-0477(1997)078<2539:GPAYMA>2.0.CO;2

[23] Kalnay E, Kanamitsu M, Kistler R, Collins W, Deaven D, Gandin L, et al The NCEP/NCAR 40-year reanalysis project. Bull Amer Meteor Soc. 1996;77: 437–471. doi: 10.1175/1520-0477(1996)077<0437:TNYRP>2.0.CO;2

[24] Gill A. Atmosphere-ocean dynamics Vol. 30 San Diego: Academic press International Geophysics Series; 1982.

[25] Grant A, Oreamuno R, Serrano A, Vargas O. Comisión sobre la problemática de inundaciones en la vertiente Atlántica Costa Rica: Colegio Federado de Ingenieros y de Arquitectos de Costa Rica; 2004.

[26] Milliman JD, Farnsworth KL. River Discharge to the Coastal Ocean: A Global Synthesis. Cambridge: Cambridge University Press; 2011.

[27] Boyer T, Locarnini RA, Zweng MM, Mishonov AV, Reagan JR, Antonov JI, et al Changes to calculations of the World Ocean Atlas 2013 for version 2 Silver Spring: National Oceanographic Data Center; 2015.

[28] Locarnini RA, Mishonov AV, Antonov JI, Boyer TP, Garcia HE, Baranova OK, et al World Ocean Atlas 2013, Volume 1: Temperature In: Levitus S, Mishonov A, editors. NOAA Atlas NESDIS 73. Silver Spring: National Oceanographic Data Center; 2013 pp. 1–40.

[29] Zweng MM, Reagan JR, Antonov JI, Locarnini RA, Mishonov AV, Boyer TP, et al World Ocean Atlas 2013, Volume 2: Salinity In: Levitus S, Mishonov A, editors. NOAA Atlas NESDIS 74. Silver Spring: National Oceanographic Data Center; 2013 pp. 1–39.

[30] Lagerloef G, Colomb R, Le Vine D, Wentz F, Yueh S, Ruf C, et al The Aquarius/SAC-D mission: Designed to meet the salinity remote-sensing challenge. Oceanogr. 2008;21(1): 68–81.

[31] Yueh S, Tang W, Fore A, Hayashi A. Aquarius CAP Algorithm and Data User Guide. Version: 3.0. Jet Propulsion Laboratory. California: California Institute of Technology; 2014.

[32] Mesa O, Poveda G, Carvajal L. Introducción al clima de Colombia 1era ed. Medellín: Universidad Nacional de Colombia; 1997.

[33] Draper N, Smith H. Applied regression analysis 3rd Ed. New York: John Wiley; 1981.

[34] Beron-Vera FJ, Ripa P. Three-dimensional aspects of the seasonal heat balance in the Gulf of California. J Geophys Res Oceans. 2000;105(C5): 11,441–11,447. doi: 10.1029/2000JC900038

[35] Ripa P. Ajuste de datos por cuadrados mínimos. Cienc. Mar. 2002;28(1): 79–105.

[36] Trenberth K. Signal versus noise in the Southern Oscillation. Mon. Weather Rev. 1984;112(2): 326–332.

[37] Semtner AJ, Chervin RM. Ocean general-circulation from a global eddy-resolving model. J Geophys Res Oceans. 1992;97(C4): 5493–5550. doi: 10.1029/92JC00095

[38] Tokmakian R. A high resolution Ocean model with variable forcing of wind, heat, and freshwater: Initial evaluation. Int. WOCE News. 1998;32: 26–28.

[39] Matano R, Beier E, Strub P, Tokmakian R. Large scale forcing of the Agulhas variability: The seasonal cycle. J Phys Oceanogr. 2008;32(4): 1228–1241. doi: 10.1175/1520-0485(2002)032<1228:LSFOTA>2.0.CO;2

[40] Matano R, Beier E, Strub P. The seasonal variability of the circulation in the South Indian Ocean: Model and observations. J Marine Syst. 2008;74(1–2): 315–328. doi: 10.1016/j.jmarsys.2008.01.007

[41] Ruiz-Ochoa M, Beier E, Bernal G, Barton ED. Sea surface temperature variability in the Colombian Basin, Caribbean Sea. Deep Sea Res Part 1 Oceanogr Res Pap. 2012;64: 43–53. doi: 10.1016/j.dsr.2012.01.013

[42] Andrade CA, Barton ED. Sobre la existencia de una celda de circulación atmosférica sobre el Caribe y su efecto en las corrientes de Ekman del Caribe suroccidental. Bol Cient CIOH. 2013;31: 73–94.

[43] Hidalgo HG, Durán-Quesada AM, Amador JA, Alfaro EJ. The Caribbean Low-Level Jet, the Inter-Tropical Convergence Zone and precipitation patterns in the Intra-Americas Sea: A proposed dynamical mechanism. Geogr Ann Ser A. 2015;97: 41–59. doi: 10.1111/geoa.12085

[44] Morrison JM, Nowlin WD Jr. General distribution of water masses within the eastern Caribbean Sea during the winter of 1972 and fall of 1973. J Geophys Res Oceans. 1982;87(C6): 4207–4229. doi: 10.1029/JC087iC06p04207

[45] Rhein M, Kirchner K, Mertens C, Steinfeldt R, Walter M, Fleischmann-Wischnath U. Transport of South Atlantic water through the passages south of Guadeloupe and across 16° N, 2000–2004. Deep Sea Res Part 1 Oceanogr Res Pap. 2005;52(12): 2234–2249. doi: 10.1016/j.dsr.2005.08.003

[46] Smith RH. Atlantic-Caribbean exchange through Windward Passage. M.Sc. Thesis, University of Miami. 2010. Available from: http://scholarlyrepository.miami.edu/oa_theses/24/.

[47] Corredor JE. Apuntes sobre la circulación costera en el Caribe noroccidental colombiano. Bol Cient CIOH. 1981;3: 3–8.

[48] Andrade CA, Barton ED. The Guajira upwelling system. Cont. Shelf Res. 2005;25: 1003–1022. doi: 10.1016/j.csr.2004.12.012

[49] Andrade CA, Barton ED. The atmospheric Low Level Jet and the surface mesoscale circulation of the Caribbean Sea. GEOS. 2009;29(1): 85.

[50] Lonin SA, Hernández JL, Palacios DM. Atmospheric events disrupting coastal upwelling in the southwestern Caribbean. J Geophys Res Oceans. 2010;115(C06030). doi: 10.1029/2008JC005100

[51] Wang C. Variability of the Caribbean Low-Level Jet and its relations to climate. Clim Dyn. 2007;29(4): 411–422. doi: 10.1007/s00382-007-0243-z

[52] Muñoz E, Busalacchi A, Nigam S, Ruiz-Barradas A. Winter and summer structure of the Caribbean Low-Level Jet. J Clim. 2008;21(6): 1260–1276. doi: 10.1175/2007JCLI1855.1

[53] Whyte FS, Taylor MA, Stephenson TS, Campbell JD. 2008. Features of the Caribbean Low Level Jet. Int J Climatol. 2008; 28(1): 119–128. doi: 10.1002/joc.1510

[54] Amador J. The Intra-Americas Sea Low-Level Jet: Overview and Future Research. Ann NY Acad Sci. 2008;1146(1): 153–188. doi: 10.1196/annals.1446.012 19076415 10.1196/annals.1446.012

[55] Cook KH, Vizy EK. Hydrodynamics of the Caribbean Low-Level Jet and Its Relationship to Precipitation. J Clim. 2010;23: 1477–1494. doi: 10.1175/2009JCLI3210.1

[56] Poveda G, Jaramillo A, Gil MM, Quiceno N, Mantilla RI. Seasonality in ENSO-related precipitation, river discharges, soil moisture, and vegetation index in Colombia. Water Resour Res. 2001;37(8): 2169–2178. doi: 10.1029/2000WR900395

[57] Poveda G, Waylen P, Pulwarty R. Annual and inter-annual variability of the present climate in northern South America and southern Mesoamerica. Palaeogeogr Palaeoclimatol Palaeoecol. 2006;234(1): 3–27. doi: 10.1016/j.palaeo.2005.10.031

[58] Poveda G, Álvarez DM, Rueda OA. Hydro-climatic variability over the Andes of Colombia associated with ENSO: A review of climatic processes and their impact on one of the Earth’s most important biodiversity hotspots. Clim Dyn. 2010;36(11–12), 2233–2249. doi: 10.1007/s00382-010-0931-y

[59] Poveda G. La hidroclimatología de Colombia: una síntesis desde la escala inter-decadal hasta la escala diurna. Rev. Acad. Col. Cien. Exact. Fís. Nat. 2004;28(107): 201–222.

[60] Tootle GA, Piechota TC, Gutiérrez F. The relationships between Pacific and Atlantic Ocean sea surface temperatures and Colombian streamflow variability. J Hydrol. 2008;349(3–4): 268–276. doi: 10.1016/j.jhydrot.2007.10.058

[61] Gordon A. Circulation of the Caribbean Sea. J Geophys Res Oceans. 1967;72(24): 6207–6223.

[62] Petus C, Garcia-Valencia C, Thomas Y, Cesaracio M. 2007. Étude de la variabilitésaisonnière et interanuelle de la résurgence de la Guajira (Colombie) par analyse de donnéessatellitaires AMI-Wind, SeaWinds et AVHRR. Rev Télédétection. 2007;7(1-2-3-4): 143–156.

[63] Paramo J, Correa M, Nuñez S. Evidencias de desacople físico-biológico en el sistema de surgencia en La Guajira, Caribe colombiano. Rev. Biol. Mar. Oceanogr. 2011;46(3): 421–430.

[64] Rueda-Roa DT, Muller-Karger FE. The southern Caribbean upwelling system: Sea surface temperature, wind forcing and chlorophyll concentration patterns. Deep Sea Res Part 1 Oceanogr Res Pap. 2013;78: 102–114. doi: 10.1016/j.dsr.2013.04.008

