# Supplementary material for: Freshwater exchanges and surface salinity in the Colombian basin, Caribbean Sea
Source: PLoS One. 2017 Aug 4;12(8):e0182116. doi: 10.1371/journal.pone.0182116 (PMC5544217; doi:10.1371/journal.pone.0182116)
Supplement: S1 Appendix — (DOCX) [file pone.0182116.s001.docx]

**List of Acronyms**

AAIW: Antarctic Intermediate Water

CAPv3: Combined Active-Passive

CICESE: Center for Scientific Research and Higher Education of Ensenada

CLLJ Index: Caribbean Low Level Jet Index

COLCIENCIAS: Administrative Department for Science, Technology and Innovation, Francisco José de Caldas of Colombia

CONACYT: National Council of Science and Technology of Mexico

CSW: Caribbean Surface Water

DJF: December to February

ENSO: El Niño Southern Oscillation

E: Evaporation

E-P: Evaporation minus precipitation

ETESA: Panama Electrical Transmission Enterprise (Empresa de Transmisión Eléctrica S.A. de Panamá)

IDEAM: Colombian Hydrology, Meteorology and Environmental Institute (Instituto de Hidrología,

Meteorología y Estudios Ambientales de Colombia)

ITCZ: Inter Tropical Convergence Zone

JJA: June to August

Lat: Latitude

Lon: longitude

MAM: March to May

N/A: Not available

Nef: Effective Degrees of Freedom

NOAA: National Oceanic and Atmospheric Administration

ONI: Oceanic El Niño Index

P: Precipitation

PCG: Panama-Colombia Gyre

POCM-4C: Parallel Oceanic Circulation Model

PODAAC: Physical Oceanography Distributed Active Archive Center

R: Runoff

SON: September to November

SSS: Sea Surface Salinity

SUW: Subtropical Underwater

TACW: Tropical Atlantic Central Water
